# Antibiotic prescribing to inpatients in Limpopo, South Africa: a multicentre point-prevalence survey

**DOI:** 10.1186/s13756-023-01306-z

**Published:** 2023-09-16

**Authors:** Tiyani Comfort Mthombeni, Johanita Riétte Burger, Martha Susanna Lubbe, Marlene Julyan

**Affiliations:** Medicine Usage in South Africa (MUSA), North-West University Potchefstroom Campus, Potchefstroom, South Africa

**Keywords:** Antibiotic use, Antibiotic stewardship, AWaRe (Access Watch Reserve), Point prevalence survey, Limpopo, South Africa

## Abstract

**Background:**

Electronic continuous surveillance databases are ideal for monitoring antibiotic use (ABU) in hospitalised patients for antibiotic stewardship programmes (ASP). However, such databases are scarce in low-resource settings. Point prevalence surveys (PPS) are viable alternatives. This report describes ABU and identifies ASP implementation improvement areas in Limpopo Province, South Africa.

**Methods:**

This cross-sectional descriptive study extracted patient-level ABU data from patients’ files using a modified global PPS tool. Data were collected between September and November 2021 at five regional hospitals in Limpopo Province, South Africa. All patients in the wards before 8 a.m. on study days with an antibiotic prescription were included. Antibiotic use was stratified by Anatomic Therapeutic Chemical and Access, Watch, Reserve classifications and presented as frequencies and proportions with 95% confidence intervals (CI). Associations between categorical variables were assessed using the chi-square test. Cramér’s *V* was used to assess the strength of these associations.

**Results:**

Of 804 inpatients surveyed, 261 (32.5%) (95% CI 29.2–35.7) were prescribed 416 antibiotics, 137 were female (52.5%) and 198 adults (75.9%). One hundred and twenty-two (46.7%) patients received one antibiotic, 47.5% (124/261) received two, and 5.7% (15/261) received three or more antibiotics. The intensive care units had a higher ABU (68.6%, 35/51) compared to medical (31.3%, 120/384) and surgical (28.5%, 105/369) wards (*p* = 0.005, Cramér’s *V* = 0.2). Lower respiratory tract infection (27.4%, 104/379), skin and soft tissue infections (SST) (23.5%, 89/379), and obstetrics and gynaecology prophylaxis (14.0%, 53/379) were the common diagnoses for antibiotic prescriptions. The three most prescribed antibiotic classes were imidazoles (21.9%, 91/416), third-generation cephalosporins (20.7%, 86/416) and combination penicillin (18.5%, 79/416). Access antibiotics accounted for 70.2% (292/416) of prescriptions and Watch antibiotics for 29.6% (123/416) (*p* = 0.110, Cramér’s *V* = 0.1). Reasons for prescribing and treatment plans were documented in 64.9% (270/416) (95% CI 60.3–69.5) and 21.4% (89/416) (95% CI 17.3–25.3) of prescriptions, respectively.

**Conclusions:**

The study serves as a baseline for ABU surveillance at the five regional hospitals in Limpopo Province. Lack of documentation indicates poor prescribing practices; ASP should address gaps by deploying evidence-based, multifaceted and stepwise interventions.

**Supplementary Information:**

The online version contains supplementary material available at 10.1186/s13756-023-01306-z.

## Introduction

The global increase and variation in antibiotic use (ABU) [1, 2] drive the increasing global public health threat of antibiotic resistance (ABR) [3]. Even though ABR is naturally occurring, its progression is accelerated by inappropriate ABU [2, 3]. Compared to other burden of disease regions in the world, Sub-Saharan Africa (SSA) had the highest all-age death rates associated with ABR, exceeding 75 per 100,000 [1, 4]. The high ABR in SSA could be attributed to a combination of One Health domain factors, such as limited access to clean water, sanitation, and hygiene, leading to a high prevalence of priority infectious diseases such as lower respiratory tract (LRTI) and bloodstream infections [2, 5]. Factors such as poor governance, limited health financing, poverty and limited access to health services also contribute to high ABR in SSA [5, 6]. Globally, one in three hospitalised patients receives an antibiotic, however there is global variance, with low and middle-income countries having higher ABU than high-income countries [7, 8] and one in every two hospitalised patients in SSA receiving an antibiotic [1, 8, 9]. The primary cause of high ABU in SSA, in particular, can be attributed to limited hospital antibiotic stewardship programme (ASP) implementation [10–13], inappropriate prescribing (e.g., unnecessary prescribing, inappropriate selection when an antibiotic is indicated, and incorrect dosing, formulation, route of administration and treatment duration) and empirical prescribing due to a lack of or non-adherence to treatment guidelines and limited microbiological testing due to inadequate laboratory facilities, resulting in a vicious cycle of a causal-effect relationship between ABU and ABR development [1, 14, 15].

Increasing ABU necessitates measures to ensure optimal use and monitoring [2, 3, 16]. In the 2015-global action plan, the World Health Organization (WHO) recognised antibiotic stewardship as one of three pillars of a comprehensive plan for strengthening health systems and global response to antibiotic resistance, infection prevention, control, and patient medicine safety [3, 16]. Global evidence suggests that implementing hospital ASPs—a set of coordinated interventions to improve, measure, and encourage access to appropriate ABU [17]—is associated with a reduction in ABU in both hospitalised and non-hospitalised patients [10]. Antibiotic stewardship interventions are implemented by ASPs with the objectives of optimising ABU, improving patient outcomes, and lowering antibiotic resistance and healthcare costs [18]. Hospital ASPs rely on ABU surveillance data, driven from data sources such as hospital files, health insurance or pharmacy dispensing records comprising patient-level details and indications to determine current ABU levels and guide future quality improvement plans [16, 19, 20].

An electronic continuous surveillance database will be ideal for collecting ABU surveillance data to guide evidenced-based ASP interventions; however, such databases are scarce in low-resource settings [19, 21]. Without electronic continuous databases to describe ABU and prescribing quality, point prevalence surveys (PPS) are useful alternative methods [21, 22]. The PPS method is used globally to describe ABU according to the Anatomic Therapeutic Chemical (ATC) and Access, Watch, Reserve (AWaRe) classifications [23–25], develop and evaluate prescribing and patient care quality indicators, identify local clinical practice areas for ASP quality improvement plans, and evaluate ASP interventions in hospitalised patients [7, 8, 11, 24–26]. Quality indicators are objective, evidence-based healthcare measures that may be utilised with hospital administrative data to assess and monitor clinical performance and outcomes [27]. ASP interventions could control antibiotic resistance through optimal ABU when combined with ABU surveillance data and quality indicators [10, 27].

There is evidence of ASP in the South African public and private sectors [22, 28–36]; however, implementation in public sector facilities is inadequate [30, 32–35]. Where ASP has been implemented, ABU surveillance has been implemented the least often compared to policy and guideline implementation and the existence of antibiotic stewardship committees [37]. Antibiotic use surveillance data has previously been collected using the PPS method in South African public sector hospitals [22, 28, 38–40], but ABU surveillance data remain inadequate [40, 41]. There is an over-representation of tertiary hospitals in PPS from previous global [7, 8, 11, 23] and national studies [38, 40], which could distort the reported ABU. Antibiotic use in regional hospitals in South Africa, including the Limpopo Province, is sub-optimal. Earlier national studies contained data from two regional hospitals, with the highest ABU of 76.3% when compared to other levels of care: central (29.6%), tertiary (37.3%), district (43.1%) and a countrywide ABU of 33.6% [38, 40]. To contribute knowledge on ABU surveillance data, we aimed to describe ABU to inpatients according to the ATC and AWaRe classification, describe antibiotic prescribing using quality indicators and identify ASP quality improvement areas in regional hospitals in Limpopo Province, South Africa.

## Methods

### Study design and setting

A cross-sectional descriptive research design was used to collect data from hospitalised patients' files from the Limpopo Province's five regional hospitals (Letaba Hospital, Mokopane Hospital, Tshilidzini Hospital, Philadelphia Hospital, and Saint Rita’s Hospital) between 07 September and 16 November 2021. Regional hospitals in the South African public sector context are secondary care facilities that operate on a 24-h basis [42]. Each of the five regional hospitals provides specialist services in paediatrics and neonatal care, obstetrics and gynaecology, anaesthesia, surgery and orthopaedics, internal medicine, psychiatry, and family medicine to 500 000–1.5 million district residents, referral services for six to seven district hospitals, and 50 to 150 primary healthcare facilities [43]. The regional hospitals receive outreach and support from tertiary hospitals and have between 200 and 800 beds [43]. The total inpatient bed capacity for the five hospitals is 1463 [43]. Therefore, the regional hospitals used in our study served as an intermediate setting to help monitor ABU in patients admitted to public sector hospitals in Limpopo Province.

### Data collection and management

A paper-based PPS data collection tool was developed using the global PPS and WHO-PPS as the basis [44, 45], with additional patient care indicators based on a multidisciplinary, international consensus on generic inpatient quality indicators that may be used globally to evaluate the quality of antibiotic use among hospitalised patients [46].

The study population consisted of all patients admitted to the hospital ward before 8 a.m. on the day of the PPS with at least one systemic antibiotic prescribed. To prevent denominator inaccuracies, all patients admitted after 8 a.m. on the day of the survey were excluded. Due to the unstable and continual transfer of patients to the wards or other hospitals, patients admitted to the emergency care department were excluded.

On the antibiotic level, this study included antibacterials for systemic use (oral, parenteral, rectal, inhalation) and antiprotozoals (i.e., nitroimidazole derivatives) used as antibacterial agents. Topical antibiotics, antimycobacterials, antivirals and antifungals were excluded. All wards at the five study centres were included and categorised according to their activities: medical, surgical, and intensive care units. All outpatient departments were excluded.

The files of hospitalised patients with an antibiotic prescription were used as a data source for patient-level demographic and antibiotic prescription data. The demographic data collected for each patient on antibiotic therapy comprised age, weight and sex. For each antibiotic prescribed, the dose, route (i.e., oral, parenteral, rectal, inhalation), prescriber type (i.e., specialised, general practitioner), diagnosis, indication, and treatment start date were collected. The diagnosis of the prescribed antibiotics was collected by applying standardised categories adopted from the global PPS protocol [44, 45]. The source of infection was also categorised based on standardised definitions and included community-acquired infection, healthcare-acquired infection, surgical prophylaxis, medical prophylaxis, and other unclear indications. If the antibiotic indication was surgical prophylaxis, an extra variable indicating the period of surgical prophylaxis was collected. The period of surgical prophylaxis was categorised as follows: one dose, one day, and more than one day (prolonged surgical antibiotic prophylaxis (SAP)).

A set of prescribing quality indicators was also collected for each prescribed antibiotic: antibiotic written in the generic name (“yes/no”), prescribing reason documented in notes (“yes/no”), South African standard treatment guidelines (STGs) adherence (“yes/no/not assessable/no information”), antibiotic treatment plans documented (“yes/no”) and whether prescribed treatment was empirical or targeted. Additional patient care indicators adapted from a global consensus study [46] were also collected for each patient on antibiotic therapy: monitoring the administration of prescribed antibiotics, specimens collected for pathogen identification, availability of results in the patient file if a specimen was collected, switching of antibiotic therapy from intravenous to oral therapy based on clinical condition within 48 to 72 h, and dosage adjustment to renal function (assessed based on trough creatinine levels and creatinine clearance). All patient care indicators data were categorised as “no/yes/unknown/not applicable”. The final two patient care indicators were missed doses (the total number of antibiotic doses missed since prescribing) and reasons for missed doses (stock-out, hang-time or unknown).

To minimise denominator complexity caused by new admissions and discharges, ward data collection was completed on the same day. We visited surgical wards the day after elective surgical interventions were scheduled to collect data about prophylaxis in the previous 24 h. Data from each hospital had to be collected within one week and during weekdays.

### Data analysis

Data were analysed using Statistical Package for the Social Sciences (SPSS^®^) version 28. Patient demographics and hospital characteristics were reported as frequencies and proportions stratified by sex (male/female), age (adults (above 18 years)/paediatric (18 years and younger), healthcare-associated infections risk factors, bed occupancy rate, and ward type. In this study, ABU was determined at the patient level (i.e., the number of inpatients with at least one antibiotic prescribed) and at the antibiotic level (total number of prescribed antibiotics) and was stratified by ward activity, source of infection, diagnosis, ATC and AWaRe classifications. The overall, mono or combination therapy and ward activity ABUs were determined by dividing the number of patients who received an antibiotic by the total number of eligible patients (admitted). ABU by ward activity was categorised as medical, surgical and intensive care units. The antibiotic per patient ratio was determined by dividing the total number of prescribed antibiotics by the number of patients with at least one prescribed antibiotic on survey days.

ABU frequencies and proportions were classified according to ATC classification level four and patient age groups and stratified by the source of infection (i.e., community-acquired infection) and diagnoses/site of infection (i.e., LRTI). To determine commonly prescribed antibiotics, individual antibiotic agents were ranked from the highest to the lowest according to their prescribing frequency and assigned their respective ATC level 4 and AWaRe classifications. ABU frequencies and proportions were also described according to the AWaRe classification and stratified by hospital, ward activity, age, prescriber type, source of infection and diagnosis. At an antibiotic level, overall antibiotic prescribing quality indicators were described using frequencies and proportions of the "yes" choice. The "yes, no, unknown, not applicable" choices were used to determine patient-level indicators of patient care quality using frequency and proportions.

For statistical inferences, ABU, prescribing and patient care indicators were represented as frequencies and proportions with 95% confidence intervals. We used the Chi-square test with a *p*-value of 0.05 for statistical significance to assess the relationship between categorical variables (e.g., between ABU and ward activity and between Access and Watch antibiotic groups and ward activity, age group and prescriber type). For practical significance interpretation, Cramér's *V* ≥ 0.6 was deemed a strong association, 0.3–0.5 moderate, 0.1–0.2 weak and < 0.1 no association.

## Results

### Patient demographics and hospital characteristics

In the five hospitals, 804 of the 1463 available beds were occupied, resulting in a bed occupancy rate of 55.0%. In 2020, the five hospitals had 340 660 inpatient days. Two-hundred and sixty-one (261) of the 804 hospitalised patients satisfied the study's inclusion criteria (inpatients with at least one antibiotic prescription) (Table 1).

**Table 1:** Healthcare-associated infection predisposing risk factors, point bed occupancy rate and annual inpatient days of inpatients at five regional hospitals in Limpopo Province, South Africa

Hospital	Total no. of inpatients on antibiotics	Healthcare-associated infections predisposing risk factors												Bed occupancy rate			Hospital annual inpatient days
		Surgery since admission		Urinary catheter		Transfer from another hospital		Intubation		Transfer from primary healthcare		HIV positive		Available beds	Occupied	Bed occupancy rate	
	N	*n*	%	*n*	%	*n*	%	*n*	%	*n*	%	*n*	%	N	*n*	%	N
Hospital A	39	8	20.5	15	38.5	8	20.5	0	0.0	5	12.8	6	15.4	242	111	45.9	54 522
Hospital B	66	26	39.4	27	40.9	17	25.8	8	12.1	1	1.5	13	19.7	341	205	60.1	79 651
Hospital C	47	8	17.0	15	31.9	0	0.0	0	0.0	14	29.8	3	6.4	268	119	44.4	70 654
Hospital D	63	9	14.3	19	30.2	14	22.2	0	0.0	12	19.1	11	17.5	346	210	60.7	79 495
Hospital E	46	8	17.4	16	34.8	18	39.1	2	4.4	17	37.0	4	8.7	266	159	59.8	56 338
Overall	261	59	22.6	92	35.3	57	21.8	10	3.8	49	18.8	37	14.2	1463	804	55.0	3 40 660

*HIV* human immunodeficiency virus, *n* frequency, *N* population

Baseline demographic data are shown in Table 2. Most patients were female (52.5%, 137/261) and adults (above 18 years) (75.9%, 198/261) (Table 2). The study population had the following healthcare-associated infection risk factors: urinary catheters (35.3%, 92/261), surgery since admission (22.6%, 59/261), or were transferred from another hospital (21.8%, 57/261) (Table 1).

**Table 2:** Characteristics of inpatients admitted at Limpopo Province regional hospitals by sex and age

Hospital	Patients admitted	Patients on antibiotics		Sex				Age			
				Female		Male		Paediatric		Adults	
								(18 years and below)		(Above 18 years)	
	N	*n*	%	*n*	%	*n*	%	*n*	%	*n*	%
Hospital A	111	39	35.1	15	38.5	24	61.5	6	15.4	33	84.6
Hospital B	205	66	32.2	38	57.6	28	42.4	13	19.7	53	80.3
Hospital C	119	47	39.5	28	59.6	19	40.4	16	34.0	31	66.0
Hospital D	210	63	30.0	36	57.1	27	42.9	20	31.8	43	68.2
Hospital E	159	46	28.9	20	43.5	26	56.5	8	17.4	38	82.6
Total	804	261	32.5	137	52.5	124	47.5	63	24.1	198	75.9

*n* frequency, *N* population

### Overall antibiotic use

Table 3 presents a summary of the overall ABU; of the 804 admitted patients, 261 were prescribed at least one antibiotic, giving an ABU of 32.5% (95% CI 29.2–35.7), with a total of 416 antibiotic prescriptions, yielding an antibiotic per patient ratio of 1.6: 1. One hundred and twenty-two (46.7%) patients received one antibiotic, compared to 124 (47.5%) on two and 15 (5.7%) on three or more antibiotics. Of the five study sites, Hospital C had the highest ABU at 39.5% (47/119) (95% CI 30.7–48.3) and Hospital E had the lowest ABU at 28.9% (46/159) (95% CI 21.9–35.9). The intensive care units had a higher ABU (68.6%, 35/51) compared to the medical (31.3%, 120/384) and surgical (28.5%, 105/369) wards (Table 4). Overall, there was a statistically significant relationship between ABU and ward type (*p* = 0.000), with a weak association between the two variables (Cramér’s *V* = 0.2) (Table 4). In terms of medical activity wards, Hospital B had the lowest ABU (15/103, 14.6%), and Hospital C had the highest ABU (28/58, 48.3%). Hospital E had the lowest ABU (13/78, 16.7%), and Hospital B had the highest ABU (40/89, 44.9%) in surgical activity wards.

**Table 3:** Overall antibiotic use among inpatients admitted at Limpopo Province regional hospitals

Hospital	Overall antibiotic us				Antibiotic per patient ratio		Antibiotic use					
	Total no. of patients admitted	Number of patients on antibiotics	Patients on antibiotic use (5)	95% CI	Number of antibiotic prescriptions (N)	Antibiotic per patient ratio	(Mono- and combination therapy)					
							One antibiotic		Two antibiotics		Three or more antibiotics	
	N	*n*	%	%			*n*	%	*n*	%	*n*	%
Hospital A	111	39	35.1	25.3–44.0	64	1.6	18	46.2	17	43.6	4	10.2
Hospital B	205	66	32.2	25.8–38.6	101	1.5	33	50.0	31	47.0	2	3.0
Hospital C	119	47	39.5	30.7–48.3	75	1.6	21	44.7	24	51.1	2	4.3
Hospital D	210	63	30.0	23.8–36.2	99	1.6	33	52.4	24	38.1	6	9.5
Hospital E	159	46	28.9	21.9–35.9	77	1.7	17	37.0	28	60.9	1	2.2
Total	804	261	32.5	29.2–35.7	416	1.6	122	46.7	124	47.5	15	5.7

*CI* confidence interval, *N* population, *n* frequency

**Table 4:** Antibiotic use inpatients at five regional hospitals of Limpopo Province by ward activity

Hospital	Total patients admitted	Number of patients on antibiotics	Medical			Surgical			Intensive care unit			*P*-value	Cramér’s *V*
			Admitted	Antibiotic use		Admitted	Antibiotic use		Admitted	Antibiotic use			
	N	*n*	N	*n*	%	N	*n*	%	N	*n*	%		
Hospital A	111	39	39	18	46.2	68	17	25.0	4	4	100.0	0.002	0.336
Hospital B	205	66	103	15	14.6	89	40	44.9	13	11	84.6	0.000	0.429
Hospital C	119	47	58	28	48.3	54	17	31.5	7	2	28.6	0.159	0.176
Hospital D	210	63	114	33	23.9	80	18	22.5	16	12	75.0	0.000	0.290
Hospital E	159	46	70	26	37.1	78	13	16.7	11	7	63.6	0.001	0.301
Total	804	261	384	120	31.3	369	105	28.5	51	35	68.6	0.000	0.204

*N* population, *n* frequency

### Antibiotic use according to the Anatomical Therapeutic Chemical classification level 4, indications and diagnosis

The most common indication for antibiotic prescriptions was community-acquired infection (55.5%, 231/416), followed by healthcare-acquired infection (17.8%, 74/416) and surgical prophylaxis (14.2%, 59/416) (Table 5). The majority of macrolides (88.9%, 16/18), three-quarters (77.2%, 61/79) of combination penicillin and two-thirds (66.3%, 57/86) of third-generation cephalosporins (3GCs) prescriptions were for community-acquired infection. All prescriptions for carbapenems (*n* = 13) were indicated for healthcare-acquired infection. The most prevalent antibiotic classes in SAP were extended-spectrum penicillins (31.0%, 22/71) and imidazole derivatives (29.7%, 27/91), whereas the majority (94.7%, 18/19) of sulphonamides-trimethoprim combinations were prescribed for medical prophylaxis (Table 5), for adults with human immunodeficiency virus infection (Additional file 1: Table S1). Community-acquired infection was the most common indication in both adults (191/321, 59.6%) and paediatrics (42.1%, 40/95) (Additional file 1: Table S1). The second common indication was healthcare-acquired infection in adults (17.1%, 55/321) and medical prophylaxis in paediatrics (32.6%, 31/95) (Additional file 1: Table S1).

**Table 5:** Antibiotic use for inpatients by indication and ATC classification

ATC classification level 4	HAI		CAI		SAP		MP		Unknown		Total	
	*n*	%	*n*	%	*n*	%	*n*	%	*n*	%	N	%
Aminoglycosides	3	8.8	15	44.1	0	0.0	16	47.1	0	0.0	34	100.0
Beta-lactam combination penicillin	14	17.7	61	77.2	4	5.1	0	0.0	0	0.0	79	100.0
Carbapenems	13	100.0	0	0.0	0	0.0	0	0.0	0	0.0	13	100.0
Glycopeptides	3	100.0	0	0.0	0	0.0	0	0.0	0	0.0	3	100.0
Imidazoles	11	12.1	53	58.2	27	29.7	0	0.0	0	0.0	91	100.0
Macrolides	1	5.6	16	88.9	0	0.0	0	0.0	1	5.6	18	100.0
Oxazolidinones	1	100.0	0	0.0	0	0.0	0	0.0	0	0.0	1	100.0
Penicillins (excluding combination penicillins)	5	7.0	28	39.4	22	31.0	16	22.5	0	0.0	71	100.0
Fluoroquinolones	1	100.0	0	0.0	0	0.0	0	0.0	0	0.0	1	100.0
Sulphonamide-trimethoprim combination	0	0.0	1	5.3	0	0.0	18	94.7	0	0.0	19	100.0
3GCs	22	25.6	57	66.3	6	7.0	0	0.0	1	1.2	86	100.0
Total	74	17.8	231	55.5	59	14.2	50	12.0	2	0.5	416	100.0

*3GC* third-generation cephalosporin, *ATC* Anatomical Therapeutic Chemical, *CAI* community-acquired infection, *HAI* healthcare-acquired infection, *MP* medical prophylaxis, *SAP* surgical antibiotic prophylaxis,* n* frequency, *N* population

Table 6 presents the ten common diagnoses for antibiotic prescriptions and their antibiotic classes (ATC level 4). LRTIs (27.4%, 104/379), SST (23.5%, 89/379), and obstetrics and gynaecology prophylaxis (14.0%, 53/379) were the three most common diagnoses for antibiotic prescriptions. Overall, imidazoles (22.7%, 86/379) were the most frequently prescribed antibiotic class in the top 10 common diagnoses list, followed by 3GCs (19.5%, 74/379) and combination beta-lactam penicillins (18.7%, 71/379). Combination penicillin (32.7%, 34/104), 3GCs (28.9%, 30/104), and macrolides (13.5%, 14/104) were the most frequently prescribed antibiotic classes for LRTI. Combination penicillins (30.3%, 27/89), imidazoles (27.0%, 24/89) and 3GCs (24.7%, 22/89) were the most frequently prescribed antibiotic classes for skin and soft tissue (SST) diagnosis. For prophylaxis in obstetrics and gynaecology, nearly half (49.1%, 26/53) of prescribed antibiotics were imidazoles, and extended-spectrum penicillin contributed two-fifths (41.5%, 22/53) of the antibiotic prescriptions. Adults had the same top three diagnoses as the total sample, while paediatrics had medical prophylaxis in neonates (42.3%, 30/71), LRTI (26.8%, 19/71), and sepsis (15.5%, 11/71) (Additional file 1: Table S1).

**Table 6:** Antibiotic use for the top ten diagnoses among inpatients, by ATC classification

Diagnosis	LRTI		SST		Proph OBGY		Neo-MP		GI		HIV		OBGY		Sepsis		CNS		BJ		Total	
Antibiotic ATC classes	*n*	%	*n*	%	*n*	%	*n*	%	*n*	%	*n*	%	*n*	%	*n*	%	*n*	%	*n*	%	N	%
Aminoglycosides	4	3.8	4	4.5	0	0.0	16	50.0	3	12.5	0	0.0	0	0.0	2	11.1	1	8.3	0	0.0	30	7.9
Beta-lactam combination penicillin	34	32.7	27	30.3	1	1.9	0	0.0	3	12.5	0	0.0	2	11.1	3	16.7	0	0.0	1	9.1	71	18.7
Carbapenems	1	1.0	2	2.2	0	0.0	0	0.0	0	0.0	0	0.0	0	0.0	8	44.4	1	8.3	0	0.0	12	3.2
Glycopeptides	0	0.0	0	0.0	0	0.0	0	0.0	0	0.0	0	0.0	0	0.0	2	11.1	1	8.3	0	0.0	3	0.8
Imidazoles	12	11.5	24	27.0	26	49.1	0	0.0	13	54.2	0	0.0	8	44.4	1	5.6	0	0.0	2	18.2	86	22.7
Macrolides	14	13.5	0	0.0	0	0.0	0	0.0	0	0.0	0	0.0	1	5.6	0	0.0	0	0.0	0	0.0	15	4.0
Oxazolidinones	0	0.0	0	0.0	0	0.0	0	0.0	0	0.0	0	0.0	0	0.0	0	0.0	1	8.3	0	0.0	1	0.3
Extended spectrum penicillins	8	7.7	10	11.2	22	41.5	16	50.0	3	12.5	0	0.0	4	22.2	0	0.0	0	0.0	5	45.5	68	17.9
Sulphonamide-trimethoprim combination	1	1.0	0	0.0	0	0.0	0	0.0	0	0.0	18	100.0	0	0.0	0	0.0	0	0.0	0	0.0	19	5.0
3GCs	30	28.8	22	24.7	4	7.5	0	0.0	2	8.3	0	0.0	3	16.7	2	11.1	8	66.7	3	27.3	74	19.5
Total	104	100.0	89	100.0	53	100.0	32	100.0	24	100.0	18	100.0	18	100.0	18	100.0	12	100.0	11	100.0	379	100.0

*3GC* third-generation cephalosporin, *ATC* Anatomical Therapeutic Chemical, *BJ* bone and joint, *CNS* central nervous system, *GI* gastrointestinal, *HIV* human immunodeficiency virus, *LRTI* lower respiratory tract infection, *Neo-MP* neonatal medical prophylaxis, *OBGY* obstetrics and gynaecology, *Proph OBGY* prophylaxis obstetrics and gynaecology, *SST* skin and soft tissue, *n* frequency, *N* Population

### Commonly prescribed antibiotic classes and agents according to the Anatomical, Therapeutic Chemical classification

The top three antibiotic classes prescribed were imidazoles (21.9%, 91/416), 3GC (20.7%, 86/416) and combination penicillins (18.5%, 79/416) (Table 5). The overall five commonly prescribed antibiotic agents (ATC level 5) were metronidazole (21.9%, 91/416), ceftriaxone (20.7%, 86/416), amoxicillin with an enzyme inhibitor (18.5%, 77/416), ampicillin (12.7%, 53/416) and gentamycin (7.2%, 30/416) (Table 7). The top five antibiotics prescribed to children and adults were similar, except for gentamycin (28.0%, 23/82) and meropenem (8.5%, 7/82) in paediatrics and sulphamethoxazole-trimethoprim (6.6%, 18/272) in adults (Additional file 1: Table S1).

**Table 7:** Frequency ranking of antibiotics prescribed for inpatients at five regional hospitals in Limpopo Province

Position	Antibiotic agent	Prescribing frequency	%	AWaRe classification	ATC classification
1	Metronidazole	91	21.9	Access	Imidazoles
2	Ceftriaxone	86	20.7	Watch	3GCs
3	Amoxicillin with an enzyme inhibitor	77	18.5	Access	Beta-lactam combination penicillin
4	Ampicillin	53	12.7	Access	Extended-spectrum penicillins
5	Gentamycin	30	7.2	Access	Aminoglycosides
6	Sulphamethoxazole-Trimethoprim	19	4.6	Access	Sulphonamide-trimethoprim combination
7	Azithromycin	18	4.3	Watch	Macrolides
8	Cloxacillin	13	3.1	Access	Extended-spectrum penicillins
9	Meropenem	11	2.6	Watch	Carbapenems
10	Amikacin	4	1.0	Access	Aminoglycosides
11	Amoxicillin	4	1.0	Access	Extended-spectrum penicillins
12	Vancomycin	3	0.7	Watch	Glycopeptides
13	Piperacillin with an enzyme inhibitor	2	0.5	Watch	Beta-lactam combination penicillin
14	Ciprofloxacin	1	0.2	Watch	Fluoroquinolones
15	Ertapenem	1	0.2	Watch	Carbapenems
16	Imipenem	1	0.2	Watch	Carbapenems
17	Linezolid	1	0.2	Reserve	Oxazolidinones
18	Phenoxymethylpenicillin	1	0.2	Access	Beta-lactamase sensitive penicillins
	Total	416	100.0		

*3GC* third-generation cephalosporin, *ATC* Anatomical Therapeutic Chemical, *AWaRe* Access Watch Reserve

### Antibiotic use by 2021 World Health Organization AWaRe classification

The antibiotic prescribing pattern according to the WHO AWaRe classification is summarised in Table 8. Access antibiotics accounted for 70.2% (292/416) of prescribed antibiotics, while Watch antibiotics accounted for 29.6% (123/416). Access antibiotics were prescribed at a relatively high proportion in surgical wards (77.8%, 133/171), while Watch antibiotics were prescribed at a relatively high proportion in medical wards (37.4%, 70/187). A statistically significant relationship was found between ward activity and the prevalence of Access and Watch antibiotics (*p* = 0.005), with a weak association between the variables (Cramér’s *V* = 0.2). Paediatric patients were prescribed a higher proportion of Access antibiotics (79.0%, 75/95), while adult patients were prescribed a higher proportion of Watch antibiotics (32.1%, 103/321). The relationships between age groups and the prescribing prevalence of Access and Watch antibiotics were statistically significant *(p* = 0.000), with a strong association between the variables (Cramér’s *V* = 1.0).

**Table 8:** Antibiotic use among inpatients at five regional hospitals in Limpopo Province, stratified by the AWaRe classification

Variables	AWaRe category	Access		Watch		Reserve		Total		*P*-value	Cramér’s *V* test
		*n*	%	*n*	%	*n*	%	N	%		
Hospital	Hospital A	45	70.3	18	28.1	1	1.6	64	100.0	0.110	0.135
	Hospital B	76	75.3	25	24.8	–	–	101	100.0		
	Hospital C	58	77.3	17	22.7	–	–	75	100.0		
	Hospital D	60	60.6	39	39.4	–	–	99	100.0		
	Hospital E	53	68.8	24	31.2	–		77	100.0		
	Total	292	70.2	123	29.6	1	0.2	416	100.0		
Ward activity	Medical	117	62.6	70	37.4	–	–	187	100.0	0.005	0.160
	Surgical	133	77.8	37	21.6	1	0.6	171	100.0		
	ICU	42	72.4	16	27.6	–	–	58	100.0		
Age group	Paediatrics	75	79.0	20	21.0	–	–	95	100.0	0.000	1.000
	Adults	217	67.6	103	32.1	1	0.3	321	100.0	0.037	1.102
Prescriber type	General	200	73.0	74	27.0	–	–	274	100.0	0.102	0.080
	Specialist	92	64.8	49	34.5	1	0.7	142	100.0		
Indication	HAI	31	41.9	42	56.8	1	1.4	74	100.0	–	–
	CAI	158	68.4	73	31.6	–	–	231	100.0		
	SP	53	89.3	6	10.2	–	–	59	100.0		
	MP	50	100.0	–	–	–	–	50	100.0		
	Unknown	–	–	2	100.0	–	–	2	100.0		
Diagnosis	LRTI	59	56.7	45	43.3	–	–	104	100.0	–	–
	SST	65	73.0	24	27.0	–	–	89	100.0		
	Prophy OBGY	49	92.5	4	7.6	–	–	53	100.0		
	Neo-MP	32	100.0	–	–	–	–	32	100.0		
	GI	22	91.7	2	8.3	–	–	24	100.0		

*AWaRe* Access Watch Reserve, *CAI* community-acquired infection, *GI* gastrointestinal, *HAI* healthcare-acquired infection, *LRTI* lower respiratory tract infection, *MP* medical prophylaxis, *Neo-MP* neonatal medical prophylaxis, *Prophy OBGY* prophylaxis obstetrics and gynaecology, *SP* surgical prophylaxis, *SST* skin and soft tissue, *n* frequency, *N* population

### Quality antibiotic prescribing and patient care indicators

Indicators for antibiotic prescribing and the quality of patient care are presented in Table 9. One-third (32.9%, 137/416) of antibiotic prescriptions were written using the generic name. Most (87.7%, 365/416) (95% CI 84.6–90.9) antibiotic prescriptions were parenteral formulations. A third (33.9%, 20/59) (95% CI 21.8–46.0) of surgical prophylaxis antibiotic prescriptions were prolonged beyond 24 h. The national STGs were adhered to in 93.3% (388/416) (95% CI 90.9–95.7) of prescriptions. Reasons for prescribing were documented in 64.9% (270/416) (95% CI 60.3–69.5) of antibiotic prescriptions, while the documentation of antibiotic treatment plans (stop/review date) was 21.4% (89/416) (95% CI 17.5–25.3). Only 3.4% (14/416) (95% CI 1.6–5.1) of antibiotic prescriptions targeted specific pathogens.

**Table 9:** Antibiotic quality prescribing and patient care indicators among inpatients in regional hospitals in Limpopo Province

Quality indicators	N	Hospital A		Hospital B		Hospital C		Hospital D		Hospital E		Overall		95% CI
		*n*	%	*n*	%	*n*	%	*n*	%	*n*	%	*n*	%	%
*Prescribing quality indicators*														
Non-proprietary prescribing (Yes)	416	20	31.5	35	34.7	22	29.3	32	32.3	28	36.4	137	32.9	28.4–37.4
Parenteral route of administration	416	52	81.3	86	85.2	69	92.0	89	89.9	69	80.6	365	87.7	84.6–90.9
Prescribing reason on notes (Yes)	416	39	60.9	60	59.4	54	72.0	59	59.6	58	75.3	270	64.9	60.3–69.5
Prolonged surgical prophylaxis	59	0	0.0	10	41.7	4	44.4	6	85.7	–	–	20	34.5	21.8–46.0
Adherence to treatment guidelines (Yes)	416	51	79.7	96	95.1	73	97.3	95	94.0	73	94.8	388	93.3	90.9–95.7
Treatment plan or stop/review date (Yes)	416	5	7.8	18	17.8	10	13.3	21	21.2	35	45.5	89	21.4	17.5–25.3
Targeted prescribing (Yes)	416	6	9.4	–	–	3	4.0	4	4.0	1	1.3	14	3.4	1.6–5.1
Patient care quality indicators														
Specimen collection (Yes)	261	8	20.5	–	–	11	23.4	9	14.3	3	6.5	31	11.9	8.0–15.8
Culture results available (Yes)	31	6	75.0	–	–	2	18.2	5	55.6	1	33.3	14	45.2	27.6–62.7
Prescribed antibiotics administered (Yes)	261	38	97.4	64	96.8	45	95.7	60	95.2	43	93.4	250	95.7	93.3–98.2
Missed doses (*n*)		5		13		7		7		9		41		
Intravenous to oral switch (No)	261	1	2.6	5	7.6	7	14.9	7	11.1	16	34.8	36	13.8	9.6–18.0
Renal dose adjustment (No)	261	–	–	2	3.0	6	12.8	1	1.6	8	17.4	17	6.5	3.5–9.5
Therapeutic drug monitoring (No)	261	2	5.1	1	1.5	2	4.3	1	1.6	–	–	6	2.3	0.5–4.1

*CI* confidence interval, *n* frequency, *N* population

Diagnostic specimens were taken in only 11.9% (31/261) (95% CI 8.0–15.8) of the patients, whereas results were available in 45.2% (14/31) (95% CI 27.6–62.7) of the cases. However, 95.7% (250/261) (95% CI 93.5–98.2) of patients were current on antibiotic therapy, totalling 41 missing doses. Thirty-six of 261 patients (13.8%) (95% CI 9.6–18.0) were identified for probable intravenous to oral switch therapy, whereas 17 patients (6.5%) (95% CI 3.5–9.5) were identified for renal dose adjustment, and six patients (2.5%) (95% CI 0.5–4.1) were identified for therapeutic drug monitoring.

## Discussion

This study used an adapted PPS tool to describe ABU in five public sector regional hospitals in Limpopo Province, South Africa, as a baseline for future comparison. Antibiotics were prescribed to approximately one-third (32.5%) of patients, which is similar to earlier South African public sector studies (31.0% and 33.6%) [39, 40]. The controlled public sector medicine procurement that implements national STGs may explain the similarities between ABU in our study and earlier South African studies [39, 47]. The ABU in this study was also compared to global trends in hospitalised adults (34.4%) and paediatrics (36.7%) [7, 8].

One hundred and twenty-two (46.7%) patients received one antibiotic, 124 (47.5%) received two, and 15 (5.7%) received three or more antibiotics. Carefully chosen combination therapy is useful, and reporting it may indicate ASP intervention areas [22, 38–40].

In our study, the intensive care unit ABU was the highest (68.6%), followed by medical (31.5%) and surgical (28.5%) wards. A similar trend was observed in a Western Cape Province tertiary hospital [39]. The intensive care unit antibiotic ABU (68.6%) in our study is consistent with the global prevalence (70.0%) [48]. We did not investigate the existence, functionality, or effect of ASP interventions. Therefore, it is unclear what contributed to the variation in ABU between the five study sites and ward activity [10]. Globally, 54.0% of intensive care unit patients are suspected or confirmed to be infected with Gram-negative organisms, associated with a 30.0% mortality [48], which may explain the high ABU in intensive care units in this study. Consequently, ASPs should promote the timely selection and administration of appropriate antibiotic therapy and de-escalation of empiric broad-spectrum antibiotics to reduce adverse patient outcomes [49].

Similar to global trends [8], the most common indication for antibiotic prescriptions in our study was community-acquired infections, followed by healthcare-acquired infections and surgical antibiotic prophylaxis, with variability between adults and paediatrics. Most prescriptions of broad-spectrum antibiotics were prescribed for community-acquired infections in accordance with the national STG [47].

Overall LRTIs, SST, obstetrics and gynaecology prophylaxis were the most common diagnoses for antibiotic prescriptions. This was comparable to previous South African studies [22, 39]. Globally, LRTIs, SST, and sepsis were the most common diagnostic reasons for antibiotic prescriptions in hospitalised patients [8, 11]. The high prevalence of LRTI diagnoses in this study corroborates global trends [50, 51]. In this study, LRTI antibiotic therapy mainly comprised a combination penicillin, 3GCs, and macrolides and is in line with national treatment guidelines [47, 52, 53].

Globally, including in South Africa and Limpopo Province, traffic accidents, self-harm, and interpersonal violence are the leading causes of injury to SST [50, 54, 55]. These injuries may result in contaminated open wounds and cellulitis that require antibiotic treatment, which is consistent with the findings of this study, demonstrating that SST was the second most common diagnosis for ABU [54, 55]. The South African STGs recommend using beta-lactams as empirical treatment for SST due to their good activity against *Staphylococcus aureus* and *Streptococcus* species [47]. In this study, combination penicillins were the most prescribed antibiotic class in SST. The second antibiotic class for SST was imidazole derivatives, which is concerning as anaerobic cultures were not typically isolated in SST in South Africa, and not aligned to the South African STGs [47, 56].

Imidazoles were the frequently prescribed antibiotic class, followed by 3GCs and combination penicillins, correlating with global trends [2, 8]. The imidazole derivatives were also common antibiotic classes in SSA countries indicated parenterally for SST and obstetrics and gynaecology prophylaxis [9, 47]. In our study, imidazoles were prescribed mainly for prophylaxis in obstetrics and gynaecology, followed by SST and gastrointestinal infections in accordance with the South African STG [47].

The five commonly prescribed antibiotic agents were metronidazole, ceftriaxone, amoxicillin with an enzyme inhibitor, ampicillin and gentamycin, which is consistent with earlier South African studies [38–40]. The extensive prescribing of 3GCs (i.e., ceftriaxone) in this study is concerning due to the high 3GCs resistance (70.0%) in bloodstream infections and *Klebsiella pneumonia* predominance in South Africa [57]. Meropenem was in the top five paediatric antibiotics (possibly for sepsis [21]), which is concerning given the high prevalence (80.0%) of carbapenem-resistant *Acinetobacter baumannii* in South Africa [57].

According to the WHO's 2021 AWaRe classification [23], 70.2% of prescribed antibiotics in our study were from the Access group. This is consistent with local antibiotic sales data [58]. The Watch ABU in our study was 29.2%, similar to national findings in adults (30.0%) and paediatrics (27.8%) [38, 40]. Ward activity and age group were statistically associated with ABU of Access and Watch antibiotics.

Antibiotic prescribing quality indicators are important for identifying the appropriateness of prescribing and establishing and monitoring the effectiveness of ASP interventions [7]. Parenteral route antibiotic prescriptions (87.7%) in this study was higher than global trends (~ 72–79%) [7, 8] and early South African studies ranging from 46 to 76% [22, 38, 40]. The high parenteral antibiotic prescribing in our study requires ASPs to develop and implement early intravenous to oral switch therapy guidelines (particularly for amoxicillin with an enzyme inhibitor and metronidazole) to minimise risks associated with catheter-related healthcare-acquired infections, high healthcare costs, and extended length of hospital stay [59–62].

In our study, there were 59 prescriptions for SAP, of which 34.5% were prolonged (> 24 h), which is lower than findings from early South African adult (73.2%) and paediatric (66.7%) studies [38, 40]. Prolonged SAP is a global concern in terms of inappropriate use for the prevention of surgical site infections [63]. The preventative efficacy of SAP is widely demonstrated in international guidelines [64]; however, prolonged SAP does not provide an advantage to surgical site infection care and may increase the risk of adverse events such as acute renal injury and *Clostridium difficile* infection; hence, an area for persuasive prospective audit and feedback ASP interventions to reduce SAP[10, 65, 66].

The adherence to antibiotic prescribing to clinical guidelines is associated with improved patient outcomes, including decreased mortality, length of stay, improved quality and lower costs in the direction of targeted prescribing [67, 68]. The adherence to national STGs was 93.3% in our study, which was similar to findings from previous public sector South African studies (90.2% and 98.0%) [22, 39] and higher than global benchmark (77.4%) [8]. The controlled public sector medicine procurement using national STGs and essential medicines lists may explain the high adherence to national STGs [47].

The documentation of antibiotic prescribing reasons was 64.9%, which is lower than the global benchmark (76.9%) [8] and findings at a Western Cape Province tertiary hospital (86.0%) [39], but comparable to hospitalised adults (64.3%) nationwide [40]. In this study, 32.9% of antibiotic prescriptions were written using non-proprietary names. The documentation of antibiotic reasons and prescribing by their non-proprietary name facilitates communication between healthcare professionals and enables the monitoring of treatment plans [8, 16]. The documentation of antibiotic treatment plans was 21.4%, double the 11.0% from a Western Cape Province tertiary hospital [39], but below the global benchmark (38.3%) [8]. The treatment plan prescribing indicator promotes therapy review within 48 to 72 h to avoid inappropriate (extended period, appropriate agent and route of administration) ABU[8]. Adherence to treatment plan documentation was low in our study and could be an area for a persuasive educational and administrative ASP intervention to encourage practice change [16, 69].

In our study, the proportion of antibiotic prescriptions targeting specific pathogen(s) was 3.4%, lower than global levels (9.8% to 22.3%) [7, 8, 26], as well as previous South African studies (8.3% to 28.8%) [38, 40]. The low-targeted prescribing observed together with low specimen collection (11.9%) and fewer results (45.2%) in our study may be attributable to a lack of point-of-care diagnostics and inadequate laboratory capacity, which may result in inappropriate diagnosis and antibiotic therapy [1, 14, 15]. 

Our study revealed additional areas for clinical ASP interventions, including intravenous to oral switch therapy, renal-dose adjustment, and therapeutic drug monitoring, which are considered advanced and costly ASP interventions [16]. Finally, our study commenced on 7 September 2021, at the tail-end of the Delta wave of the Coronavirus disease of 2019 (COVID-19) pandemic in South Africa and ended on 16 November 2021, before the Omicron variant wave began (23 November 2023) [70]; however, ABU was similar to pre-pandemic studies in South Africa [40]. Modelling of global antibiotic sales data shows an increase of 0.3% per 1000 individuals for a 10% increase in COVID-19 cases [71]. Given that our study was conducted between two COVID-19 infection waves [70], we believe that the pandemic may not have had a significant influence on the ABU during our study period.

The following limitations were noted in this study. A PPS is limited by its cross-sectional study design since data were collected over a short period; this approach did not account for seasonal variation in diseases and antibiotic prescriptions. A more accurate estimate of inpatient ABU may have been obtained by conducting this survey over several periods using serial or a seasonal repeated PPS. This study was conducted in regional (secondary) hospitals, and its findings cannot be generalised to other levels of care (primary or tertiary hospitals) as global and national evidence suggests ABU heterogeneity by hospital type [8, 38, 40]. Our findings cannot be generalised to the private sector settings, considering that antibiotic prescribing and STGs adherence vary between the two sectors in South Africa [72].

## Conclusion

The study serves as a baseline for ABU surveillance at the five regional hospitals in Limpopo Province. Lack of documentation indicates poor prescribing practices; ASP should address gaps by deploying evidence-based, multifaceted and stepwise interventions. [16, 73].

### Supplementary Information

**Additional file 1**. **Table S1:** Antibiotic use among inpatients at five regional hospitals in Limpopo Province, stratified by patient age.

## Abbreviations

ATC: Anatomic Therapeutic Chemical
3GC: Third-generation cephalosporin
ABU: Antibiotic use
ASP: Antibiotic Stewardship programme
AWaRe: Access, Watch, Reserve
COVID-19: Coronavirus disease of 2019
LRTI: Lower respiratory tract infection
PPS: Point prevalence survey
SAP: Surgical antibiotic prophylaxis
SSA: Sub-Saharan Africa
SST: Skin and soft tissue
STG: Standard treatment guideline
WHO: World Health Organization
